# Impact of Credit Constraints from Formal Financial Institutions on Rural Residents’ Health in China

**DOI:** 10.3390/healthcare9010006

**Published:** 2020-12-23

**Authors:** Fan Yang, Yao Jiang, Krishna P. Paudel

**Affiliations:** 1 Department of Labor and Social Security, School of Public Administration, Sichuan University, Chengdu 610065, China; yangfan1987@scu.edu.cn; 2 Department of Accounting, College of Management, Sichuan Agricultural University, Chengdu 611130, China; s20175706@stu.sicau.edu.cn; 3 Department of Agricultural Economics and Agribusiness, Louisiana State University (LSU) and LSU AgCenter, Baton Rouge, LA 70803, USA

**Keywords:** credit constraints, health, rural resident, poverty, China

## Abstract

This is the first study in China that looks at the impact of credit constraints from formal financial institutions on Chinese rural residents’ health. We use the Chinese Household Income Project (CHIP) data collected by the Annual Household Survey Office of Integration of Urban and Rural in the National Bureau of Statistics in 2014. We measure rural residents’ health status with self-rated health assessment and the number of sick days in 2013. The results obtained from using the ordered probit model show that, in general, credit constraints from formal financial institutions significantly and negatively affect the self-rated health of Chinese rural residents. When an endogeneity issue is addressed using the instrumental variable (IV) approach, this paper’s results are still robust. The results also show that the impact of credit constraints from formal financial institutions on rural residents’ self-rated health is significant in male, female, married, and unmarried sub-groups. Further, we find that credit constraints from formal financial institutions impact rural residents’ health through income and economic vulnerability. The findings have implications for preventing rural residents from falling into a health trap due to credit constraints from formal financial institutions.

## 1. Introduction

This paper uses the most recent Chinese Household Income Project (CHIP) data collected by the Annual Household Survey Office of Integration of Urban and Rural in the National Bureau of Statistics in 2014. We use the ordered probit model and the instrumental variable (IV) ordered probit model to establish the link between Chinese rural residents’ self-rated health status and the credit constraint they face from formal financial institutions. For sensitivity analyses, we also use the reported number of illness days in a year to replace self-rated health and probe how it is affected by credit constraints from formal financial institutions.

As a basic human capital, health is the carrier and premise of other human capital forms, such as knowledge and skills [1]. Health has an impact on individuals’ productivity and quality of life. According to recent statistics released by the Chinese government, the proportion of rural poverty caused by illness is more than 42% in China, and more than 40% of these poor rural residents are between 15 and 59 years old [2]. Instead of becoming a productive member of society, they need to be tended to and cared for. When these poor people get sick, they drain household resources on health care costs and directly affect other family members’ productivity. Poor health makes poor rural residents even poorer. It is of great significance to identify the influencing factors of rural residents’ health to terminate the link among “health influencing factors-health impairment-poverty” for poverty reduction in China’s rural areas.

At present, the research on the influencing factors of individual health mainly focuses on the individual demographic attributes [3,4,5,6], the social attributes and behaviors of individuals [7,8,9], and the impact of environmental quality and hazards [10,11,12,13,14,15,16]. These papers presented different perspectives and laid a foundation for empirical research on individual health. However, little attention has been paid to the impact of credit constraints on individual health.

Our paper focuses on the impact of formal credit constraints on Chinese rural residents’ health. The credit constraints from formal financial institutions are such that a rural resident cannot get the desired amount of credit at a market interest rate and on time from the formal financial institutions [17]. With the advancement of a series of rural reform measures since 1979, the financial market in Chinese rural areas has developed substantially. By the end of 2018, the total number of formal financial institutions in rural areas was 126,600. The average number of bank outlets in a county was 56.41, and the average number of bank outlets in a township was 3.95 [18]. The primary formal financial institutions providing loans in rural areas are the Agricultural Development Bank of China, the Postal Savings Bank of China, the Rural Credit Cooperatives, and the Agricultural Bank of China [19].

Still, rural residents face severe formal credit constraints due to the lack of valid collateral and information asymmetry, such as unfamiliarity with the process of credit application. The land titling policy implemented between 2013 and 2018 has alleviated the Chinese rural credit problem to some extent [20]. Chinese farmers (also known as peasants because of their small land holding size) started getting land titling certificates effective 2013. Before 2013, collectives had land ownership rights and farmers had land management rights. Farmers were not allowed to officially transfer (rent) their land management rights to others. However, the small and fragmented land holdings do not foster land renting easily in the formal market. Credit constraints hinder rural residents from investing in productive capital [21], restrict their income growth and consumption level [22], and inhibit rural residents’ welfare increase [23]. Indeed, the impact of credit constraints on rural residents goes beyond these explicit aspects. This paper argues that credit constraints also have a significant negative impact on rural residents’ health.

This paper aims to investigate the impact of formal credit constraints on rural residents’ health in China. Our main contributions are as follows: first, by examining the impact of formal credit constraints on rural residents’ health, we contribute to two strands of literature: credit constraints and individual health. Second, this study clarifies the mechanism of credit constraints on rural residents’ health theoretically and empirically. Third, this study provides a decision-making reference for the government to improve rural residents’ health policy from credit constraints and credit supply improvement. This study is the first attempt to empirically analyze the impact of credit constraints on rural residents’ health in China to the best of our knowledge.

The remainder of this paper is organized as follows. Section 2 provides a theoretical framework for analyzing the impact of credit constraints on individual health. In Section 3, we detail the methods used in the paper. In Section 4, we report the results and discuss the findings. In Section 5, we provide conclusions and discuss the policy implications of the findings.

## 2. Theoretical Framework

Why people face credit constraints can be interpreted from the two aspects of credit demand and credit supply. From the credit demand perspective, the primary theoretical basis of credit constraints is the theory of credit demand repression [24]. Loan rejection rates are higher due to transaction costs and stringent credit conditions set forth by formal financial institutions [25,26,27], which results in subjective abandonment or the failure of loan applications by some borrowers, thus resulting in credit constraints.

From the credit supply perspective, the primary theoretical basis of credit constraints is credit rationing theory [28]. Under the condition of fixed interest rates, facing adverse selection and moral hazard caused by information asymmetry [29,30], banks are forced to adopt some non-interest-rate loan conditions, such as handling fees, transportation fees, and time involvement [31]. These non-interest-rate loan conditions will pull some fund demanders out of the bank loan market [32]. As a result, these people are subject to credit constraints.

Credit constraints restrict the promotion of individual human capital [33], reduce individual productivity [34], reduce income [35], limit health care [36], and increase the economic vulnerability of individuals [37]. Specifically, the impact of credit constraints on individual health is realized in the following three aspects. First, credit constraints impact individual income, which in turn impacts individual health. Second, credit constraints impact individual health by affecting individual consumption. Third, credit constraints can impact individual economic vulnerability, which in turn impacts individual health.

First, credit constraints hinder individuals from increasing their income and reduces their ability to invest in health, thereby affecting their health. Dong et al. found that under credit constraints, production inputs purchase and education for children cannot be fully funded, hindering agricultural productivity and income increase [38]. Li and Zhang [39] explored the impact of credit constraints on rural residents’ income based on the present value maximization model of rural residents’ expected income. The results show that credit constraints have a significant negative impact on the income of 10%, 25%, and 90% quantile of rural residents. Li and Sun found that formal financial credit constraints reduce rural resident’s productive and operational income by 16.1%, informal financial credit constraints reduced rural resident’s productive and operational income by 13.5%, and both formal and informal financial credit constraints reduced rural resident’s productive and operational income by 19.1% [23].

Specifically, the impact mechanism of credit constraints on individual income includes the following two aspects. On the one hand, credit constraints hinder the promotion of individual human capital and decrease individual income. Although early studies do not find the significant impact of credit constraints on human capital formation, the significant impact of credit constraints on human capital has been shown over time [21,40,41]. The evolution of this conclusion is not the choice of estimation methods but a real empirical phenomenon. Li et al. found that families encountering credit constraints have a significant negative impact on their children’s number of years in education [42]. Education can not only increase personal income but also increase its non-market effects on individuals, such as the awareness and investment of health and nutrition, thus affecting individual health.

Credit constraints reduce the production, technology adoption, operation, and income of a family [43,44,45]. In addition to affecting current output, credit constraints also have an investment demand effect, viz., reducing the ability to anticipate and formulate long-term plans [45,46]. Furthermore, credit constraints also affect rural residents’ income by changing allocation efficiency. When rural residents have more land and less labor, credit constraints make it impossible to hire laborers to carry out farm activities efficiently [27]. In entrepreneurship behavior, credit constraints affect resource allocation and rural residents’ entrepreneurship ability [47].

Second, credit constraints affect an individual’s consumption and investment in health. According to Modigliani’s life cycle theory and Friedman’s permanent income hypothesis, households can smooth consumption expenditure through intertemporal resource allocation [48,49]. Due to income instability, the lack of credit guarantee, information asymmetry, and an imperfect credit system, rural household credit constraints in developing countries are more severe than in the developed countries [22,30,36,50,51,52,53]. Lack of sufficient food causes stunting, wasting, malnutrition, and diseases, and a household’s inability to borrow to smooth out consumption restriction will perpetuate these effects.

Third, credit constraints can affect individuals’ health by affecting their economic vulnerability. Economic vulnerability refers to the economic capacity to withstand damages caused by adverse events [54]. The impact of economic vulnerability on health is mainly realized on three fronts: (1) economic vulnerability will bring more significant economic pressure to individuals or families, which will affect individuals’ or family members’ mental state and thus affect their health [55]. (2) Economic pressures brought about by economic vulnerability will force individuals or family members to engage in long work hours. Long hours of work and overtime, as an unhealthy lifestyle, will damage their health to some extent [56,57,58]. (3) Economic vulnerability is characterized by a low ability to cope with uncertainties. When faced with sudden illness or diseases, individuals or families may not be able to pay for treatment, resulting in long-term chronic effects [59].

**Figure 1:**
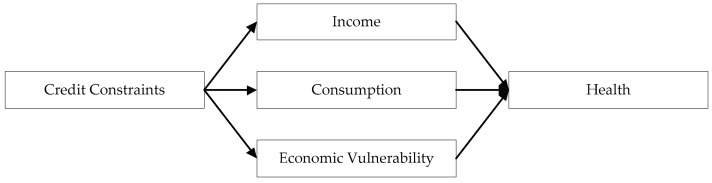
Paths of credit constraints impacting health.

To sum up, credit constraints impact individual health by affecting their income, consumption, and economic vulnerability (Figure 1). In the latter part of this paper, we will test the mechanism through empirical analysis.

## 3. Methods

### 3.1. Data

The data used in this paper come from the survey data of the CHIP collected in July and August 2014. The survey was supported by the National Natural Science Fund and National Bureau of Statistics and organized by China Institute for Income Distribution in Beijing Normal University. The survey was conducted by the Annual Household Survey Office of Integration of Urban and Rural in the National Bureau of Statistics. The survey was stratified according to the eastern, central, and western regions of China. Samples were collected by the systematic sampling method covering 18,948 household samples and 64,777 individual samples selected from 234 counties (districts) of 126 cities in 15 provinces, including 7175 urban household samples, 11,013 rural household samples, and 760 migrant worker household samples. The data include household income, expenditure, household members’ personal information, labor time arrangement, employment information, household assets, demolition and land acquisition, agricultural operation, credit constraints, and health [60]. In this paper, the final observations consisted of 21,991 rural residents aged 18–65 years.

### 3.2. Measurements

#### 3.2.1. Dependent Variable

The specific definitions of variables and descriptive statistics are shown in Table 1.

The dependent variable in this paper is the rural residents’ health. Accurate measurement of individual health is not easy. This paper adopted the approach used by existing research [61,62,63], viz., using rural residents’ subjective evaluation of health (in other words, self-rated health) to measure their health. Survey participants were asked, “How do you evaluate your current health?” The answer was measured by the five-point Likert scale ranging from “1” to “5”, which separately indicates that health status is very unhealthy, somewhat unhealthy, normal, somewhat healthy, or very healthy. An answer of “very unhealthy” was coded as “1”, “somewhat unhealthy” as “2”, “normal” as “3”, “somewhat healthy” as “4”, and “very healthy” as “5”.

Additionally, this paper also used “the number of days that the respondent could not work or live a normal life due to illness in 2013 (illness days)” as an objective measure of health status. It is a continuous variable—the greater its value, the worse the health of the respondent.

#### 3.2.2. Explanatory Variable

**Figure 2:**
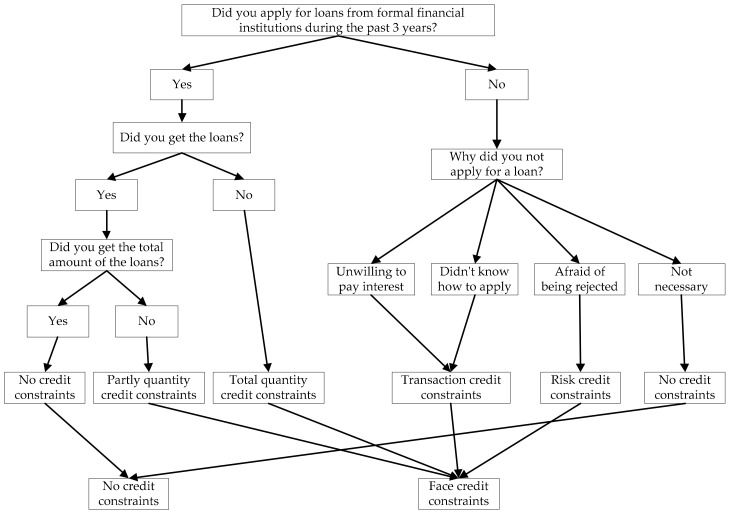
Assessing if an individual faces credit constraints from formal financial sources.

The explanatory variable in this paper was rural residents’ credit constraints with formal financial institutions. We used the method provided by Li et al. to assess whether a rural resident suffers from formal credit constraints [22]. Figure 2 displays the specific evaluation process of formal credit constraints. If a respondent suffers from formal credit constraints, the credit constraint value is “1”; otherwise, it is equal to “0”.

#### 3.2.3. Control Variables

In addition to the formal credit constraint variable, many other factors also affect individual health. Our control variables included individual characteristics and family characteristics variables. We also control the regional effects using province-level dummy variables.

Individual characteristic variables included gender, age, ethnicity, marriage, education, health insurance, and family rank. Gender may influence individual health differently through the family division of labor, employment, working conditions, and career trajectory [64]. It is a dummy variable. Male was coded as “1”, female is coded as “0”. Generally speaking, the older the age, the greater the health concerns [65]. Age is a continuous variable. Individuals in different ethnic groups have different socio-cultural contexts. Some minorities in China hold a fatalistic attitude to disease, which may deteriorate their health status [66]. Therefore, ethnicity may be a factor affecting health. In this paper, it is a dummy variable. Han ethnicity (the majority of Chinese people) was coded as “1” while other ethnicities were coded as “0”. Marriage can bring risk-sharing and emotional support to individuals, which is conducive to better health [67]. It is a dummy variable in this paper. Married was coded as “1”, while unmarried as “0”. Generally, education brings health awareness and health management ability [68,69]. Therefore, education should be a factor affecting health, and it is a continuous variable. Individuals who purchased insurance have more confidence in coping with future health risks [70]. Therefore, health insurance should be a factor affecting individual health. It is a dummy variable. A respondent with health insurance was recorded as “1”; otherwise, they were recorded as “0”. When resources are limited, parents may implement unbalanced resource allocation among their children—the first child may get the most care. Therefore, ranking among siblings may have different effects on individual health [71]. In this paper, family rank is a dummy variable. If the respondent was the first child of a family, the value was coded as “1”; otherwise, the value was coded as “0”.

The family characteristic variables include family size, per capita household income (PCHI), per capita household consumption (PCHC), per capita household debt (PCHD), and household economic vulnerability (HEV). To a certain extent, family size impacts the health of family members, which is reflected in two opposite aspects. Large family size can bring more assistance from family members to help individuals who experience diseases and maintain health than a smaller family size, while in the case of limited family resources, individuals in big families are allocated fewer health resources and have worse health than those in small families [72]. Therefore, family size is a potential factor affecting health, and it is a continuous variable. Previous studies have shown that family economic factors impact individual health [59,73,74]. In this paper, PCHI is a continuous variable measured by the logarithm of per capita household income in 2013. Similarly, PCHC and PCHD are also continuous variables and separately measured by the logarithm of per capita household consumption in 2013 and the logarithm of per capita household debt in 2013. For the HEV, survey respondents were asked, “How would you think of your family’s financial ability to cope with the risk of a variety of health situations?” The answer was measured by a four-point Likert scale ranging from “1” to “4”. The answer of “my family’s financial ability is capable of dealing with almost all risk of a variety of health situations” was coded as “1”, the answer of “my family’s financial ability is capable of dealing with the most risk of a variety of health situations” was coded as “2”, the answer of “my family’s financial ability is capable of dealing with some risk of a variety of health situations” was coded as “3”, the answer of “my family’s financial ability is incapable of dealing with the risk of a variety of health situations” was coded as “4”.

### 3.3. Empirical Model

The dependent variable, self-rated health of rural residents, is an ordered discrete variable, so we use an ordered probit regression model to do the analyses [75]. As such, a conceptual model can be written as follows:

$$y_{i}=f\left(x_{i},Z_{i}\right)$$

where $y_{i}$ is the dependent variable, representing the self-rated health of rural resident i; $x_{i}$ represents the formal credit constraints status of rural resident i; and $Z_{i}$ represents control variables that may affect the health of rural resident i. The empirical model is formulated as:

$$y^{*}=\beta x+\gamma Z+\varepsilon$$

We do not observe $y^{*}$; however, we observe y

$$y=\left\{\begin{array}{c} =1\;if\;y^{*}\leq 1\\ =2\;1<y^{*}\leq \mu_{1}\\ =3\;\mu_{1}<y^{*}\leq \mu_{2}\\ \cdots \;\cdots \;\\ =J\;y_{i}^{*}\geq \mu_{J-2} \end{array}\right.$$

where $\mu_{1}<\mu_{2}<\cdots <\mu_{J-2}$ are unknown parameters that must be estimated along with β and γ. $y^{*}$ is an unobservable variable referred to as a latent variable. Endogenous problems between rural residents’ self-rated health and formal credit constraints are present in this model. The reasons are as follows:

First, there may be a selection bias associated with formal credit constraints faced by rural residents. Formal credit constraints result from rural residents’ self-selection, such as not applying for loans, which is affected by unobservable factors. At the same time, these unobservable factors may have an impact on rural residents’ health. For example, rural residents with higher livelihood capital (social or human) are less likely to encounter formal credit constraints because they have more collaterals. At the same time, rural residents with more capital maintain their health, so they are more likely to be healthy. Therefore, rural residents’ formal credit constraints are not the result of a random selection but endogenous selection.

Second, there may be a mutually causal relationship between formal credit constraints and health. As previously analyzed in the theoretical framework section, formal credit constraints may affect individual health through income, consumption, and economic vulnerability. Meanwhile, health may also affect individual credit access through credit ratings.

Third, there may be a problem of data measurement biases on self-rated health. Due to subjective judgment limitations, it is difficult to reflect individuals’ health levels by self-rated health. Therefore, there may be measurement bias in the measurement of rural residents’ health.

Fourth, there may be some unobservable factors that also can affect credit constraints and health. In addition, although we have included variables that may affect health as control variables in the model, there may still be the problem of missing variables.

Therefore, considering that adopting traditional measurement methods results in biases in the estimated results, how to solve the endogenous problems between the two variables, self-rated health and formal credit constraints, has a direct bearing on the study’s reliability. To solve the endogeneity problem, this paper uses the IV ordered probit model for estimation [76,77].

Based on the peer effect, we choose the proportion of respondents, except for individual i, who are subject to formal credit constraints in a county as the IV. The proportion of respondents, except for individual i, who are subject to formal credit constraints in a county fulfills two basic requirements of IV: relevance and exclusion. The rural residents in the same county, to no small extent, face a similar formal credit environment and experience the same loan application process, which indicates that IV in this paper is closely related to the formal credit constraints of rural residents. However, the proportion of respondents except for individual i who are subject to formal credit constraints in a county has no direct relationship with rural residents’ health, meeting the exclusion restriction. Specifically, the IV can be written as follows:

when individual i is subject to formal credit constraints: $IV=\frac{n-1}{N-1}$

when individual i is not subject to formal credit constraints: $IV=\frac{n}{N-1}$

Here, n is the proportion of respondents who are subject to formal credit constraints in a county, and N represents the total number of respondents in a county.

## 4. Results and Discussion

### 4.1. Descriptive Statistics

It can be observed from Table 1 that the mean value of the dependent variable, rural residents’ self-rated health, is 4.0413, ranging from “1” (very bad) to “5” (very good), which indicates that the majority of Chinese rural residents believe they are in good health. Meanwhile, on average, respondents’ illness days are 6.8426 days, with a minimum of 0 days and a maximum of 365 days.

The explanatory variable, credit constraints, has an average value of 0.2702, which means 27.02% of rural residents have experienced formal credit constraints in the past three years before the interview.

Concerning individual characteristics, out of the total respondents, 52.13% of the subjects are male and 47.87% are female, showing that the proportion of males and females in the samples is approximately equal. The ages of all samples range from 18 to 65 years old, with an average age of 40.9860 years. Han ethnicity people comprise 91.90% of the total respondents, while 8.1% of respondents are of other ethnicities, which is in line with China’s actual situation. According to China’s sixth population census conducted in 2010, the Han population in China comprised 92% of the total, and while the proportion of other ethnic groups was 8% [78]. Of the respondents, 78.19% are married, whereas 21.81% of respondents are not. The respondents’ average length of schooling is 8.0376 years, which is consistent with the reality of nine-year compulsory education in China. Of the respondents, 77.88% have purchased health insurance, while 22.12% of respondents have not. Of the respondents, 38.42% are the first child in a family, and 68.58% are not.

The mean value of family size is 4.3233, with a minimum of 1 and a maximum of 13. The logarithm of per capita household income family is 9.1700, the logarithm of per capita household consumption is 8.6184, and the logarithm of per capita household debt 1.7450. The mean value of household economic vulnerability is 2.8406, ranging from 1 to 4.

Before the regression analysis, we conducted a multicollinearity test on the variables. The results show that the average value of the variance inflation factor (VIF) of the variables was 1.33, the maximum value of VIF was 2.04, and the minimum value of VIF was 1.03, which means that there is no serious multicollinearity between variables.

### 4.2. Benchmark Regression

First, an ordered probit model is estimated as a benchmark model, and the results are reported in Table 2. Column (1) of Table 2 reports the influence of formal credit constraints on rural residents’ self-rated health estimated by using an ordered probit regression model without other control variables. As can be observed from column (1), the formal credit constraints variable influences rural residents’ self-rated health significantly and negatively at a 1% level. The results presented in column (2) show that the negative influence of formal credit constraints on rural residents’ self-rated health is still statistically significant at a 1% level after controlling for regional fixed effects. Column (3) shows that the formal credit constraints have a statistically significant and negative influence on rural residents’ self-rated health at a 1% level once controlling the regional fixed effects (province) and respondents’ characteristics. After adding the regional fixed effects, respondents’ characteristics, and family characteristics variables into the ordered probit model, the results in column (4) reveal that the negative influence of formal credit constraints on rural residents’ self-rated health is still statistically significant at a 1% level.

For the control variables, gender, marriage, education, health insurance, family size, and PCHI are positively and significantly associated with rural residents’ self-rated health. This means that compared with females, rural male residents have higher self-rated health levels. Similarly, previous have found that men are less likely to suffer from diseases than women [79,80]. Compared with unmarried individuals, marriage is more beneficial to self-rated health. The result is consistent with the study of Robles and Kiecolt-Glaser [81]. The self-rated health level increases with the improvement of rural residents’ education level. Dilmaghani came to a similar conclusion in a study on the causal effects of education on health [82]. Buying health insurance is conducive to self-rated health. As analyzed in the previous section, individuals with health insurance have more confidence in coping with future health risks [70]. The larger the family size, the higher the self-rated health level of rural residents, meaning that family size plays a positive role in resisting health risks in Chinese rural households. Per capita household income is a positive factor affecting rural residents’ self-rated health. More income means a strong ability to invest in health.

Age and HEV relate to rural residents’ self-rated health negatively and significantly. Almost self-evident, age is a depreciation factor in health. With the increase in age, everyone’s health will gradually deteriorate until death. Family economic vulnerability is a negative factor affecting health. This result is consistent with our previous theoretical expectations. To sum up, the effects of control variables are mostly in line with our theoretical expectations and consistent with previous studies.

The above results can be summarized as follows: formal credit constraints reduce the probability that a rural resident judges their health condition as good and increases the probability that a rural resident judges their health condition as bad. To conclude, the benchmark regression results are consistent with our theoretical expectation; that is, formal credit constraints negatively affect rural residents’ health. However, the benchmark regression does not solve the endogenous problem. Therefore, in the next part, we add IV to solve the endogenous problem and show the negative causal relationship between formal credit constraints and rural residents’ health.

### 4.3. Solving the Problem of Endogeneity

To solve the endogenous problems mentioned in the empirical model section, this paper uses the IV two-stage least square (IV-2SLS) model and the IV ordered probit model for estimation [83]. The results, reported in Table 3, show that after controlling the endogenous problem, the impact of formal credit constraints on rural residents’ self-rated health remains statistically significant. Specifically, in the 2SLS model, the formal credit constraints variable significantly and negatively impacts the respondents’ self-rated health at a 1% level, and the coefficient is −0.6762. Similarly, in the IV ordered probit model, the formal credit constraints variable also significantly and negatively impacts the respondents’ self-rated health at a 1% level, and the coefficient is −0.7543. The results after dealing with the endogenous problems are consistent with those of the benchmark regression. In conclusion, formal credit constraints significantly reduce respondents’ self-assessment of their health.

We conducted a diagnostic test of the IV. The F-value is greater than 10, which indicates that the IV used in this article, formal credit constraints ratio of rural residents in the same county except for the respondent *i*, is a strong IV. We also find a high R^2^ value in the first stage regression, and the coefficient is significant. When we included IV in the health to credit constraint regression, the IV is insignificant. These results satisfy the relevance and excludability conditions [32].

Signs of control variables are still consistent with those of the benchmark regression. In particular, gender, marriage, education, health insurance, family size, and PCHI positively and significantly impact rural residents’ self-rated health, while age and HEV impact rural residents’ self-rated health negatively and significantly.

### 4.4. Robustness Test

#### 4.4.1. Sub-Sample Regression

To investigate the heterogeneity of the impact of formal credit constraints on Chinese rural residents’ health with different characteristics, we have estimated sub-sample regression models using an IV ordered probit model. Based on gender and marriage, we divide the sample into four sub-groups (male, female, married, and unmarried) to regress the impacts of formal credit constraints on rural residents’ self-rated health. The results are reported in Table 4. The results of the sub-sample regression models are similar to those of the whole sample. That is, formal credit constraints significantly reduce the self-rated health of male, female, married and unmarried rural residents, meaning that formal credit constraints have a negative impact on rural residents’ health.

Furthermore, based on PCHI, we divide the sample into four sub-groups (PCHI < 25%, 25% ≤ PCHI < 50%, 50% ≤ PCHI < 75%, PCHI ≥ 75%) to regress the impacts of formal credit constraints on rural residents’ self-rated health. The results are reported in Table 5. The results of the sub-sample regression models are also similar to those of the whole sample. That is, formal credit constraints significantly reduce the self-rated health of different income groups, meaning that formal credit constraints have a negative impact on rural residents’ health.

#### 4.4.2. Alternate Dependent Variable

To overcome the bias associated with self-rated health classifications, the variable of “the number of days that respondent cannot work and live normally due to illness in 2013 (illness days)” is applied to estimate the impact of formal credit constraints on rural residents’ health. Because the illness days are a limited explanatory variable with a minimum value of 0, the Tobit model is suitable for estimation [84]. The Tobit and IV-Tobit regression results are displayed in Table 6.

It can be observed from Table 6 that after controlling the variables of individual characteristics, family characteristics of respondents, and regional fixed effects (province), the coefficient of formal credit constraints is positive and significant at a 1% significance level. To overcome the endogeneity concern in the regression model, an IV–Tobit regression model is estimated. The IV used is the formal credit constraint ratio of rural residents in the same county except for the respondent *i*. As Table 6 shows, after adding IV into the regression model, the coefficient associated with the variable formal credit constraints is still positive and significant at a 1% significance level. Overall, the results of the Tobit and IV–Tobit model estimation in Table 6 indicate that formal credit constraints do have a negative impact on rural residents’ health, and our results shown in Table 3 are thus robust.

### 4.5. Mechanism Analysis

The bootstrap estimation procedure is followed to test the significance of respondents’ mediating effects of income, consumption, and economic vulnerability. Bootstrap samples are drawn 100 times to test the mediating effects. The 95% confidence intervals (CIs) of the direct and indirect effects are shown in Table 7. It can be seen that the Credit constraints→Consumption→Health model pathway is not significant. Instead, we find that the variables of income and economic vulnerability mediated the effect of formal credit constraints and health.

## 5. Conclusions

This paper used China Household Income Project data collected by the Annual Household Survey Office of Integration of Urban and Rural in the National Bureau of Statistics in 2014 to assess the impact of formal credit constraints on Chinese rural residents’ health. Results using an ordered probit model show that, in general, formal credit constraints significantly and negatively affected the self-rated health of Chinese rural residents. The results are consistent when endogeneity concerns were corrected in the model. An alternate objective measure for health status was chosen as a dependent variable to overcome the bias associated with self-assessed health evaluation. In this case, the dependent variable was “the number of days that a respondent was not able to work due to illness in 2013.” The results show that the conclusions of this paper are still robust. The results also show that the impact of formal credit constraints on rural residents’ self-rated health was significant in male, female, married and unmarried sub-groups. We found that income and economic vulnerability played a mediating role in the impact of formal credit constraints on Chinese rural residents’ health.

Health is a fundamental human right and an indicator of poverty. Formal credit constraints have a severe negative impact on individuals’ health. Therefore, the practical implication of this paper is that removing the formal credit constraints faced by rural residents will be conducive to maintaining rural residents’ health. As able bodies are likely to work, the poverty rate among healthy adults should go down.

As for how to remove the formal credit constraints of rural residents, we provide some suggestions. The first is to loosen the credit constraints in rural areas by establishing more formal/governmental financial institutions. Most of the formal financial institutions in China’s rural areas are located in towns, but some rural residents live far away from the town. They have limited knowledge about the availability and formal credit application process. Therefore, formal financial institutions should make rural residents aware of the loan availability and loan application process.

The second suggestion is to encourage rural residents to borrow from formal/governmental financial institutions. Rural residents lack valid collateral, are unwilling to pay additional interest, and lack the confidence to apply for loans successfully. Therefore, building confidence is crucial. On this issue, through the professional guidance of non-governmental organizations, setting a successful example can play an exemplary role.

Third, there should be government officers advising/training rural residents to choose profitable enterprises. The most critical thing is that formal financial institutions should allow rural residents to borrow money, and government departments and social public welfare organizations should help rural residents to operate/select profitable crop/livestock enterprises, to realize a virtuous cycle of “loan-production-loan repayment.”

There is still room for further study on this topic. For example, this paper focuses on the impact of formal credit constraints on the health of Chinese rural residents who do not have that much access to formal credit. Informal credit plays a vital role in many daily activities, including borrowing for illness treatment. Future discussions should consider overall credit access in rural areas rather than only access to formal credit. Collecting data from the same household over time should help us capture household dynamics, including the effects of credit constraints on the household’s health status.

## Figures and Tables

**Table 1:** Definition of variables and descriptive statistics (*n* = 21,991).

Variable	Definition	Mean	SD	Min	Max
*Dependent variables*					
Self-rated health	1 = very unhealthy; 2 = somewhat unhealthy; 3 = normal; 4 = somewhat healthy; 5 = very healthy	4.0413	0.8426	1	5
Illness days	The number of days that respondent cannot work due to illness in 2013	6.8426	31.1678	0	365
*Variable of interest*					
Formal credit constraints	1 = experienced formal credit constraints; 0 = no formal credit constraints	0.2702	0.4441	0	1
*Control variables*					
Gender	1 = male; 0 = female	0.5213	0.4996	0	1
Age	Years	40.9860	13.4870	18	65
Ethnicity	1 = Han ethnicity; 0 = otherwise	0.9190	0.2728	0	1
Marriage	1 = married; 0 = otherwise	0.7819	0.4130	0	1
Education	Years of schooling	8.0376	3.1301	0	21
Health insurance	1 = health insurance has been purchased; 0 = otherwise	0.7788	0.4151	0	1
Family rank	1 = the first child of a family; 0 = otherwise	0.3842	0.4864	0	1
Family size	The number of members in the household	4.3233	1.4435	1	13
PCHI	The logarithm of per capita household income in 2013	9.1700	0.7216	4.7593	12.3884
PCHC	The logarithm of per capita household consumption in 2013	8.6184	0.6835	5.5215	11.6953
PCHD	Logarithm of per capita household debt in 2013	1.7450	3.5303	0	12.7657
HEV	1–4 represents family economic vulnerability from weak to strong	2.8406	0.6365	1	4

**Table 2:** Influence of formal credit constraints on rural residents’ self-rated health (ordered probit model).

Variables	(1)	(2)	(3)	(4)
Credit constraints	−0.3094 ***	−0.2886 ***	−0.3103 ***	−0.2579 ***
	(0.0380)	(0.0369)	(0.0380)	(0.0376)
Gender			0.1021 ***	0.1079 ***
			(0.0127)	(0.0129)
Age			−0.0260 ***	−0.0257 ***
			(0.0011)	(0.0012)
Ethnicity			−0.0809	−0.1163
			(0.0904)	(0.0868)
Marriage			0.1862 ***	0.1462 ***
			(0.0265)	(0.0270)
Education			0.0438 ***	0.0375 ***
			(0.0047)	(0.0046)
Health insurance			0.1039 **	0.0903 **
			(0.0415)	(0.0405)
Family rank			0.0108	0.0125
			(0.0167)	(0.0166)
Family size				0.0298 **
				(0.0124)
PCHI				0.1057 ***
				(0.0351)
PCHC				−0.0064
				(0.0356)
PCHD				−0.0064
				(0.0046)
HEV				−0.2566 ***
				(0.0286)
Province	No	Yes	Yes	Yes
Pseudo R^2^	0.0068	0.0152	0.0665	0.0782
*n*	21,991	21,991	21,991	21,991

Notes: Robust standard errors aggregated at county level in parentheses; *** *p* < 0.01, ** *p* < 0.05.

**Table 3:** Impact of formal credit constraints on rural residents’ self-rated health IV-2SLS and IV ordered probit model.

Variables	IV-2SLS	IV-Ordered Probit
Credit constraints	−0.6762 ***	−0.7543 ***
	(0.0478)	(0.0500)
Gender	0.0857 ***	0.1124 ***
	(0.0108)	(0.0152)
Age	−0.0192 ***	−0.0257 ***
	(0.0006)	(0.0008)
Ethnicity	−0.0827 ***	−0.1173 ***
	(0.0238)	(0.0323)
Marriage	0.1239 ***	0.1379 ***
	(0.0161)	(0.0227)
Education	0.0236 ***	0.0331 ***
	(0.0023)	(0.0030)
Health insurance	0.0584 ***	0.0842 ***
	(0.0138)	(0.0198)
Family rank	0.0023	0.0096
	(0.0113)	(0.0157)
Family size	0.0183 ***	0.0268 ***
	(0.0044)	(0.0060)
PCHI	0.0535 ***	0.0807 ***
	(0.0111)	(0.0153)
PCHC	0.0033	0.0044
	(0.0115)	(0.0154)
PCHD	0.0058 ***	0.0057 **
	(0.0020)	(0.0025)
HEV	−0.1411 ***	−0.2228 ***
	(0.0090)	(0.0129)
Province	yes	Yes
Atanhrho_12		0.3287 ***
		(0.0337)
*n*	21,991	21,991
R-squared	0.1110	

Notes: Standard errors in parentheses; *** *p* < 0.01, ** *p* < 0.05.

**Table 4:** Impact of formal credit constraints on rural residents’ self-rated health on different groups as obtained from using IV-ordered probit models.

Variables	Male	Female	Married	Unmarried
Credit constraints	−0.8166 ***	−0.6855 ***	−0.7141 ***	−0.8658 ***
	(0.0663)	(0.0756)	(0.0586)	(0.0956)
Gender			0.1288 ***	0.0945 ***
			(0.0172)	(0.0342)
Age	−0.0254 ***	−0.0259 ***	−0.0265 ***	−0.0241 ***
	(0.0011)	(0.0012)	(0.0009)	(0.0019)
Ethnicity	−0.1001 **	−0.1360 ***	−0.1100 ***	−0.1533 **
	(0.0453)	(0.0461)	(0.0363)	(0.0714)
Marriage	0.1481 ***	0.1257 ***		
	(0.0310)	(0.0338)		
Education	0.0290 ***	0.0357 ***	0.0268 ***	0.0488 ***
	(0.0044)	(0.0043)	(0.0035)	(0.0061)
Health insurance	0.1052 ***	0.0610 **	0.1186 ***	0.0393
	(0.0273)	(0.0289)	(0.0243)	(0.0355)
Family rank	0.0124	0.0079	0.0119	0.0071
	(0.0218)	(0.0228)	(0.0178)	(0.0341)
Family size	0.0149 *	0.0385 ***	0.0253 ***	0.0342 **
	(0.0083)	(0.0086)	(0.0066)	(0.0142)
PCHI	0.0856 ***	0.0761 ***	0.0912 ***	0.0558
	(0.0211)	(0.0222)	(0.0171)	(0.0341)
PCHC	0.0044	0.0033	0.0173	−0.0607 *
	(0.0213)	(0.0223)	(0.0173)	(0.0347)
PCHD	0.0084 **	0.0028	0.0021	0.0182 ***
	(0.0034)	(0.0037)	(0.0028)	(0.0052)
HEV	−0.2032 ***	−0.2439 ***	−0.2462 ***	−0.1375 ***
	(0.0180)	(0.0185)	(0.0146)	(0.0277)
Province	Yes	Yes	Yes	Yes
Atanhrho_12	0.3817 ***	0.2724 ***	0.2876 ***	0.4579 ***
	(0.0462)	(0.0493)	(0.0386)	(0.0705)
*n*	11,464	10,527	17,195	4796

Notes: Standard errors in parentheses; *** *p* < 0.01, ** *p* < 0.05, * *p* < 0.1.

**Table 5:** Impact of formal credit constraints on rural residents’ self-rated health on different income groups as obtained from using IV-ordered probit models.

Variables	PCHI < 25%	25% ≤ PCHI < 50%	50% ≤ PCHI < 75%	PCHI ≥ 75%
Credit constraints	−0.7101 ***	−0.8377 ***	−0.8155 ***	−0.7853 ***
	(0.0932)	(0.1013)	(0.0947)	(0.1274)
Gender	0.0899 ***	0.1045 ***	0.1422 ***	0.1177 ***
	(0.0303)	(0.0312)	(0.0296)	(0.0306)
Age	−0.0271 ***	−0.0251 ***	−0.0268 ***	−0.0236 ***
	(0.0015)	(0.0017)	(0.0016)	(0.0017)
Ethnicity	−0.1048 *	−0.1859 ***	−0.0296	−0.1850 **
	(0.0555)	(0.0636)	(0.0636)	(0.0909)
Marriage	0.0784 *	0.0565	0.1680 ***	0.2297 ***
	(0.0446)	(0.0466)	(0.0441)	(0.0471)
Education	0.0313 ***	0.0491 ***	0.0306 ***	0.0210 ***
	(0.0059)	(0.0062)	(0.0061)	(0.0063)
Health insurance	0.0554	0.1622 ***	0.1016 ***	0.0383
	(0.0395)	(0.0412)	(0.0385)	(0.0415)
Family rank	0.0028	0.0301	0.0123	0.0263
	(0.0309)	(0.0324)	(0.0309)	(0.0321)
Family size	0.0577 ***	0.0372 ***	0.0135	0.0044
	(0.0116)	(0.0123)	(0.0116)	(0.0127)
PCHI	0.0166	−0.3203 ***	0.2823 **	0.1826 ***
	(0.0393)	(0.1193)	(0.1112)	(0.0485)
PCHC	0.0065	0.0871 **	−0.0664 **	0.0081
	(0.0307)	(0.0344)	(0.0316)	(0.0293)
PCHD	−0.0084 *	0.0137 **	0.0078	0.0113 **
	(0.0046)	(0.0055)	(0.0050)	(0.0053)
HEV	−0.2099 ***	−0.1522 ***	−0.1965 ***	−0.2874 ***
	(0.0268)	(0.0279)	(0.0249)	(0.0258)
Province	Yes	Yes	Yes	Yes
Atanhrho_12	0.3270 ***	0.3711 ***	0.3863 ***	0.3157 ***
	(0.0649)	(0.0708)	(0.0653)	(0.0814)
*n*	5493	5151	5790	5557

Notes: Standard errors in parentheses; *** *p* < 0.01, ** *p* < 0.05, * *p* < 0.1.

**Table 6:** Influence of formal credit constraints on rural residents’ illness days in 2013 (Tobit and IV-Tobit model).

Variables	Tobit	Tobit	IV-Tobit	IV-Tobit
Credit constraints	0.1915 ***	0.1195 ***	1.5438 ***	0.9406 ***
	(0.0378)	(0.0339)	(0.1673)	(0.1944)
Gender		−0.0540 ***		−0.1511 ***
		(0.0150)		(0.0453)
Age		0.0154 ***		0.0444 ***
		(0.0011)		(0.0024)
Ethnicity		−0.0379		−0.1137
		(0.1013)		(0.0912)
Marriage		−0.0902 ***		−0.0402
		(0.0253)		(0.0700)
Education		−0.0316 ***		−0.0741 ***
		(0.0044)		(0.0089)
Health insurance		−0.0252		−0.1992 ***
		(0.0449)		(0.0606)
Family rank		−0.0173		−0.0995 **
		(0.0162)		(0.0470)
Family size		−0.0128		−0.0557 ***
		(0.0111)		(0.0172)
PCHI		0.0040		0.0036
		(0.0293)		(0.0450)
PCHC		−0.0347		−0.1072 **
		(0.0336)		(0.0457)
PCHD		0.0137 ***		0.0234 ***
		(0.0044)		(0.0077)
HEV		0.1141 ***		0.2171 ***
		(0.0248)		(0.0388)
Province	No	Yes	No	Yes
Wald test			46.73 ***	11.91 ***
*n*	21,991	21,991	21,991	21,991

Notes: Standard errors in parentheses; *** *p* < 0.01, ** *p* < 0.05.

**Table 7:** Mediating effects of income, consumption, and economic vulnerability in the impact of formal credit constraints on rural residents’ self-rated health.

Model Pathways	Estimated Effect	95% Confidence Interval	
		Lower Bounds	Upper Bounds
**Direct Effect**			
(1) Income→Health	−0.1904 *** (0.0130)	−0.2133	−0.1675
(2) Consumption→Health	−0.1904 *** (0.0132)	−0.2125	−0.1683
(3) Economic vulnerability→Health	−0.1904 *** (0.0130)	−0.2127	−0.1681
**Indirect Effect**			
(1) Credit constraints→Income→Health	−0.0081 *** (0.0011)	−0.0104	−0.0058
(2) Credit constraints→Consumption→Health	0.0000 (0.0003)	−0.0004	0.0004
(3) Credit constraints→Economic vulnerability→Health	−0.0183 *** (0.0018)	−0.0214	−0.0152

Notes: Standard errors are inside parentheses. *** *p* < 0.01.

## Data Availability

Restrictions apply to the availability of these data. Data was obtained from the Center for Social Science Survey at Sun Yat-sen University in Guangzhou, China and are available from cssdata@mail.sysu.edu.cn with the permission of the Center for Social Science Survey at Sun Yat-sen University in Guangzhou, China.
